# Eight-Hour Time-Restricted Feeding: A Strong Candidate Diet Protocol for First-Line Therapy in Polycystic Ovary Syndrome

**DOI:** 10.3390/nu15102260

**Published:** 2023-05-10

**Authors:** Bihter Senem Feyzioglu, Cenk Mustafa Güven, Zerrin Avul

**Affiliations:** 1 Department of Obstetrics and Gynecology, Private Erciyes-Kartal Hospital, 38020 Kayseri, Turkey; bihtersd@yahoo.com (B.S.F.);; 2 Department of Obstetrics and Gynecology, Izmir Private Can Hospital, 35630 Izmir, Turkey

**Keywords:** polycystic ovary syndrome, eight-hour time-restricted feeding, intermittent fasting, insulin resistance, hyperandrogenism

## Abstract

We aimed to investigate the effects of a 6-week program of 8 h time-restricted feeding (TRF) diet in polycystic ovary syndrome (PCOS), as determined by anthropometric, hormonal, metabolic profiles, and fecal calprotectin level. Thirty women diagnosed with PCOS underwent a 6-week 8 h TRF diet intervention. Age, anthropometric features (body mass index (BMI), waist-to-hip ratio (WHR)) and biochemical results were recorded. Free androgen index (FAI, defining hyperandrogenism) and the homeostatic model assessment-insulin resistance (HOMA-IR) were calculated. Baseline (pre-diet) and 6-week post-diet findings were compared. Mean age was 25.57 ± 2.67 years. BMI (*p* < 0.001) and WHR (*p* = 0.001) were found to have significantly decreased after the diet, as well as the percentage of patients defined to have hyperandrogenism (*p* = 0.016). Reproductive hormone levels, FAI (*p* < 0.001) and HOMA-IR (*p* < 0.001) were improved significantly. Metabolic parameters associated with glucose and lipid profiles were also significantly improved after the diet. Additionally, fecal calprotectin levels demonstrated a significant decrease from pre-diet to post-diet (*p* < 0.001). In conclusion, a 6-week diet intervention with 8 h TRF may be a suitable and effective intermittent fasting protocol that can be used as a first-line option in PCOS.

## 1. Introduction

The most common endocrine disorder, Polycystic Ovary Syndrome (PCOS), has a prevalence ranging from 6% to 21% in premenopausal women [1,2]. Besides reproduction-related abnormalities such as menstrual disorders, infertility and hyperandrogenism, PCOS is closely associated with other comorbidities such as obesity, insulin resistance, hyperinsulinemia, and dyslipidemia [3,4]. In relation with these effects, PCOS is associated with higher risks of developing long-term complications such as diabetes and cardiovascular disease [3].

Although the exact etiology of PCOS is still unclarified [4], evidence shows that insulin resistance is crucial for hyperandrogenism, leading to detrimental effects such as chronic anovulation [4]. Obese women with PCOS, which represent around 60 of PCOS cases [5,6], have excess fat which further elevates insulin resistance [1]. The medical treatment of PCOS is limited to antiandrogenic drugs and insulin sensitizing drugs, but there is no consensus regarding their use as first-line options [2,4]. Currently, lifestyle modifications (e.g., diet and exercise) are recommended as initial therapy [7]. To date, the benefit of many diet types in PCOS management has been demonstrated [8,9,10,11,12]. Additionally, the effects of various fasting practices on PCOS comorbidities have been investigated [13,14,15], albeit in a limited number of studies.

Intermittent fasting (IF) is a general term describing alternative eating and fasting practices, including alternate-day or periodic fasting (the 5:2 or 6:1 diet), time-restricted feeding (TRF) and Ramadan-like (during-the-day) fasting [16,17,18]. Research has indicated that intermittent fasting (IF) may be as effective as, or even more effective than, continuous energy restriction with the same energy intake in terms of improving insulin sensitivity [19], and it may also have a positive impact on oxidative stress and inflammation through various pathways and lipid levels [20]. There are also studies that evaluated TRF diet in terms of glucose and lipid levels [21,22], which may be associated with oxidative stress and insulin resistance [23]. Although a few animal and human studies have reported the benefits of TRF on hormonal and metabolic disorders in PCOS [13,15], evidence that would lead to the use of this therapy for PCOS treatment is absent.

The gut microbiome has been closely linked to human metabolism, including insulin resistance [24,25]. In light of all available data, the intestinal flora and its impact on the gastrointestinal system are suggested to affect insulin resistance in PCOS [24]. Intestinal permeability, which can be influenced by altered gut flora, is one such factor implicated in insulin resistance among PCOS patients [26]. However, relationships between altered intestinal permeability and fasting therapies have not been investigated in PCOS patients.

We therefore aimed to investigate the effects of an 8 h-per-day TRF intervention as first-line treatment in PCOS. We analyzed potential effects on anthropometric parameters, hormones, lipids, and fecal calprotectin—which is a marker of increased intestinal permeability.

## 2. Materials and Methods

### 2.1. Study Plan

Ethical approval for this retrospective study was obtained from the local ethics committee of İzmir Bakırçay University (date: 17 March 2022, no: 579). The study was conducted at the Private İzmir Can Hospital, Izmir, Turkey and the Private Kayseri Erciyes Hospital, Kayseri, Turkey from August 2022 to January 2023 according to the ethical standards stated in the Declaration of Helsinki.

### 2.2. Participants

PCOS was diagnosed according to the Rotterdam criteria [27], and women with PCOS who received the TRF diet intervention as first-line treatment were reviewed retrospectively. Patients with comorbidities that could affect dietary needs or would have significantly impacted hormonal homeostasis (thyroid disease, adrenal disease, Cushing syndrome, sex hormone-secreting tumors, high prolactin, diabetes, and severe cases with heart, gastrointestinal, renal and hepatic diseases) and those reporting frequent alcohol intake or smoking were not eligible for study enrollment. Exclusion criteria were: being aged <18 and >40 years, having a body mass index (BMI) of >30 or <18 kg/m^2^, using oral contraceptives or other hormonal therapies (antiandrogens, ovulation induction agents, drugs enhancing insulin sensitivity), having received treatment with anti-epileptic or psychomotor drugs, statins and steroids within the previous 3 months, and pregnancy/lactation/pre-menopausal condition. In addition, in order to prevent confounding effects on the parameters investigated, subjects who had received antibiotic therapy within the previous 3 weeks and those with active acute infection or any gastrointestinal disease at the time of sampling were excluded.

**Figure 1:**
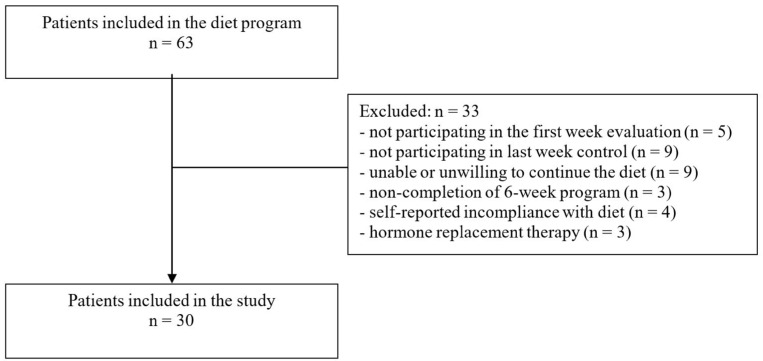
The flowchart of secondary exclusion after diet administration.

Subject to above criteria, a final group of 63 patients received TRF intervention. During assessment for data analysis, another group of patients was planned to be excluded according to following criteria: missing data, not returning for the first-week evaluation (*n* = 5) or last-week control (*n* = 9), unable or unwilling to adhere to the TRF diet (*n* = 9), non-completion of the 6-week TRF diet program due to any reason (*n* = 3), self-reported incompliance with diet (*n* = 4), and initiation of any hormone replacement therapy during the diet (*n* = 3). The remaining 30 participants were analyzed (Figure 1).

### 2.3. Laboratory Findings

Patients’ age, anthropometric measurements including BMI and waist-to-hip ratio (WHR), blood results including thyroid-stimulating hormone (TSH), prolactin, insulin, anti-Mullerian hormone (AMH), estradiol (E2), total testosterone, sex hormone-binding globulin (SHBG), dehydroepiandrosterone sulfate (DHEAS), free testosterone, serum luteinizing hormone (LH), follicle-stimulating hormone (FSH), high-density lipoprotein cholesterol (HDL-C), low-density lipoprotein cholesterol (LDL-C), triglycerides, glucose, glycated hemoglobin (HbA1c) and fecal calprotectin levels were obtained by reviewing hospital computer records and patient files.

### 2.4. PCOS Diagnosis and Management

All PCOS diagnoses were assigned in accordance with the “2018 International Evidence-based Guidelines for the Assessment and Management of PCOS” [7] and the “Rotterdam consensus criteria” proposed by the “European Society of Human Reproduction and Embryology” and the “American Society of Reproductive Medicine” [27]. Accordingly, the presence of two of the following parameters was defined as PCOS: oligo-anovulation, hyperandrogenism (clinical or laboratory findings), and presence of polycystic ovaries. Ultrasonographic measurements of the ovary were performed by experienced gynecologists using calibrated ultrasound devices (GE Voluson S10; GE, Milwaukee, WI, USA) equipped with 5–9 MHz or 6–12 MHz transducers, according to a standardized protocol [28].

### 2.5. Time-Restricted Feeding Protocol

After all women were informed in detail about the way and the possible benefits and harms of all dietary alternatives, they were allowed to make their own dietary choices and were asked to decide on their inclusion into the diet program. Written informed consent for participation into the 6-week diet program was routinely obtained from all women. The TRF diet was applied as follows: While the patients were free to eat and drink from 1 pm to 9 p.m. (8 h), they were asked to fast from 9 p.m. to 1 p.m. the next day (16 h). Participants were encouraged to drink plenty of fluids during the fasting period. However, these fluids were limited to water and calorie-free beverages in order not to reduce the possible positive effects of diet on metabolic and hormonal parameters. This diet was applied for 6 weeks, and during these 6 weeks, the participants were also asked to avoid simple carbohydrates as much as possible [13]. Hereafter, the diet used in this study will be referred to as 8 h TRF.

### 2.6. Laboratory Findings

Blood samples (venous, following 8 h of fasting) and stool samples were taken from all participants on the 3rd to 5th days of spontaneous menstrual cycles (within 3 days of diet initiation and termination). Samples were centrifuged (10 min, 1500× *g*, 4 °C) and the serum and plasma were stored at −80 °C. Serum TSH (TSH CLIA Kit; Ig Biotechnology, Burlingame, CA, USA), prolactin (Prolactin CLIA Kit; Ig Biotechnology), insulin (Insulin CLIA Kit; Ig Biotechnology), AMH (AMH CLIA Kit; MyBioSource, San Diego, CA, USA), E2 (Estradiol CLIA Kit; Ig Biotechnology), total testosterone (Testosterone CLIA Kit; Ig Biotechnology), SHBG (SHBG CLIA kit; MyBioSource), and DHEAS (DHEA-S ChLIA Kit; MP Biomedicals, Swedesboro, NJ, USA) were measured by an automated device (Siemens Medical Solutions Diagnostics; Chicago, IL, USA). Free testosterone levels were calculated using validated formulae (60). Serum LH (LH ELISA Kit; MyBioSource), FSH (FSH ELISA Kit; MyBioSource), and fecal calprotectin (Calprotectin ELISA Kit; MyBioSource) were measured by enzyme-linked immunosorbent assay (ELISA) (EL×800 ELISA reader; BioTek Instruments Inc., Winooski, VT, USA). Plasma HDL-C (HDL-Cholesterol Assay Kit—Colorimetric; AssayGenie, Dublin, Ireland), LDL-C (LDL-Cholesterol Assay Kit—Colorimetric; AssayGenie), triglycerides (Triglyceride Assay Kit—Colorimetric; AssayGenie), glucose (Glucose Assay Kit—Colorimetric; AssayGenie), and HbA1c (HbA1c Direct Enzymatic Colorimetric Kit; Atlas Medical, Istanbul, Turkey) were measured (Siemens Medical Solutions Diagnostics; Chicago, IL, USA). All laboratory measurements were carried out in the Department of Private Erciyes Kartal Hospital, Kayseri using calibrated standard measuring devices and according to the manufacturer’s recommendations.

### 2.7. Anthropometric Measurements

Anthropometric measurements were collected before and after the diet therapy. Patients removed their heavy clothing and shoes before assessments. Height was measured using a standard portable stadiometer (Seca 208; Vogel and Halke, Hamburg, Germany) and weight using a mechanical weight scale (model 160KL; Health-O-Meter Inc., Chicago, IL, USA). BMI was calculated by dividing weight (kg) by height (m) squared (kg/m^2^) [28]. Waist and hip circumference were measured and WHR was calculated in accordance with the “World Health Organization Waist Circumference Expert Consultation” [29].

### 2.8. Insulin Resistance Determination

Insulin resistance was evaluated by the homeostatic model assessment-insulin resistance (HOMA-IR) score: HOMA-IR = Fasting Glucose (mg/dL) × Fasting Insulin (uIU/mL)/405. Diagnostic cut-off was a value of ≥2.5 [30].

### 2.9. Hyperandrogenism Determination

Hyperandrogenism was determined by calculating the free androgen index (FAI) as described in [7] and with the following formula: FAI score = [total testosterone (mmol/L)/SHBG (nmol/L) × 100]. Those with a result of ≥8 were defined to have hyperandrogenism [31].

### 2.10. Statistics

All data were entered into an SPSS database (IBM, Armonk, NY, USA), and analyses were subject to the classical threshold of two-tailed *p* < 0.05. To determine whether the variables followed a normal distribution, we used the Shapiro–Wilk test. For continuous variables, we reported the data as either mean ± standard deviation or median (1st quartile–3rd quartile) based on the result of the distribution test. Categorical variables were presented as frequency (percentage). We analyzed normally distributed variables using the paired *t*-test, and non-normally distributed variables using the Wilcoxon signed ranks test. For categorical variables, we used the McNemar test.

## 3. Results

Mean age was 25.57 ± 2.67 (range 21–33) years. BMI and WHR were found to have significantly decreased after the diet (*p* < 0.001), indicating improved anthropometric results. In addition, almost all parameters associated with insulin resistance demonstrated significant improvements after the diet, including the levels of fasting insulin (*p* < 0.001), fasting glucose (*p* < 0.001) and HbA1c (*p* < 0.001), and mean HOMA-IR score (*p* < 0.001). Fecal calprotectin levels also demonstrated a significant decrease from the pre-diet to post-diet condition (*p* < 0.001).

In terms of reproductive hormone parameters, we found that the post-diet levels of AMH (*p* < 0.001), FSH (*p* = 0.002), LH (*p* < 0.001), E2 (*p* < 0.001), prolactin (*p* = 0.038), total testosterone (*p* < 0.001), free testosterone (*p* < 0.001), DHEAS (*p* < 0.001) and FAI (*p* < 0.001) were significantly lower compared to the pre-diet levels. On the other hand, post-diet TSH (*p* = 0.001) and SHBG (*p* < 0.001) were higher compared to baseline. Moreover, the percentage of patients defined to have hyperandrogenism (*p* = 0.016) was significantly lower than the pre-diet percentage.

For the lipid profile, we found that LDL-C (*p* < 0.001) and triglyceride (*p* < 0.001) levels were significantly decreased after the diet, whereas HDL-C had significantly increased compared to the baseline levels (*p* < 0.001) (Table 1).

## 4. Discussion

The effects of 8 h TRF diet, which is frequently used but lacks sufficient evidence for its potential effects in PCOS patients, was assessed for its impact on anthropometric indexes, hormonal and metabolic profiles, and intestinal permeability in PCOS patients. As a result, we found that a 6-week program of 8 h TRF provided significant improvement in all four categories.

Obesity is common in PCOS, and it can exacerbate the metabolic symptoms of the syndrome due to insulin resistance [6]. Concomitant obesity and PCOS increases cardiometabolic risks, menstrual irregularity, hyperandrogenism, hirsutism and decreased health-related quality of life [5,6]. A 5–10% decrease in body weight can have beneficial impacts on mitigating risk factors associated with cardiovascular disease, type 2 diabetes, as well as endocrine and reproductive parameters in individuals diagnosed with PCOS [32,33]. In addition, weight loss can lead to conversion of androgens to estrogen and can reduce dysregulation of crucial hormonal axes [4]. In the present study, we found that 6 weeks of 8 h TRF diet provided a significant reduction in BMI and WHR in women with PCOS. Previous studies have also shown the positive effects of fasting diets on total weight, BMI, and waist circumference [34,35]. However, data on TRF diet are very limited. In the study of Li et al., 6 weeks of 8 h TRF diet significantly improved all common anthropometric measures, but not WHR (in women with anovulatory PCOS) [13]. In a PCOS mouse model study, mice receiving 8-week ad libitum or 8 h TRF diets were compared. This diet significantly decreased body weight and reduced adiposity [15]. Regarding other fasting diets, in one study, a 6-month 5:2 diet (2 days on diet, 5 off) was shown to provide equal weight loss relative to continuous energy restriction (7 days/week) in young overweight women [36]. Considering the important relationship between anthropometric measurements and insulin resistance, and the role of insulin resistance in PCOS outcomes, it appears that TRF diet might have potential as first-line therapy in PCOS management.

Disturbances in the sex hormone levels and the irregular menstruation are the most common problems encountered in PCOS [13]. Hyperinsulinemia resulting from insulin resistance leads to an increase in androgen production by direct action and indirect effects, ultimately causing the reproductive consequences of PCOS [1,4]. Fasting may improve ovarian function by reducing glucose and insulin levels [4]. Therefore, fasting may also improve the symptoms and signs associated with hyperandrogenism in PCOS [4]. Our study demonstrated that a six-week intervention of 8 h TRF resulted in significant reductions in AMH, FSH, LH, E2, prolactin, total and free testosterone, and DHEAS levels. Additionally, the FAI score and the percentage of patients with hyperandrogenism decreased, while SHBG levels increased significantly. In a related study by Han et al., the androgen, estrogen, and LH/FSH ratio improved in PCOS mice receiving eight weeks of TRF. Histological analyses also showed lower cyst formation and improved corpus luteum formation [15]. Li et al. showed that 5 weeks of TRF significantly improved total testosterone, SHBG, and FAI score but did not alter LH, FSH levels in anovulatory PCOS; however, menstrual cycles normalized in over 70% of patients [13]. A recent review on intermittent fasting (IF) reported that it led to decreased testosterone levels and FAI scores, and increased SHBG levels. However, no effect was recorded on estrogen, gonadotropin, or prolactin. The review emphasized that IF may be a useful intervention for hyperandrogenism treatment in PCOS by improving menstrual cycles [17]. In the study by Li et al. [13], TRF diet was administered for 5 weeks, and the daily fasting time interval was between 4 pm and 8 am the following day. Previous studies have reported that SHBG contents (as well as other reproductive hormones) increase after various diets in patients with PCOS [13,17]. Of these hormones, only SHBG is important in our study, because the increased androgen and decreased SHBG levels seen in obese patients lead to higher serum concentrations of free testosterone [37]. Increasing SHBG and decreasing androgen levels is a desirable goal in PCOS management, indicating the importance of our results. It is impossible to speculate on whether the decrease in FSH and LH levels in our study was due to the difference in the overall length of the diet program (6 weeks) or the fasting time interval (from 9 p.m. to 1 p.m. the next day). However, the TRF diet appears to be able to improve the reproductive comorbidities of PCOS by disrupting the feedback mechanism that ends with hyperandrogenism. We hope that the precise effect of TRF on sex hormones in PCOS will be clarified by further studies.

Patients with PCOS are at risk of developing diabetes and heart disease due to factors such as insulin resistance, compensatory hyperinsulinemia, glucose intolerance, metabolic syndrome, and dyslipidemia, all of which have been associated with higher androgen levels [6,38,39]. These metabolic changes in PCOS may be independent of obesity, because insulin resistance is detected in 75% of lean women and 95% of obese women with PCOS [40]. It has also been reported that 70% of patients with PCOS have dyslipidemia, regardless of BMI [41]. Fasting is suggested to positively effect glucose homeostasis by altering the expression of brain-derived neurotrophic factor [16]. This has been demonstrated in animal studies, but there is insufficient evidence in humans [42]. IF diets may also benefit metabolic and endocrine disorders in PCOS by changing the circulating levels of Insulin-like Growth Factor-1 (IGF-1) and Insulin-like Growth Factor-Binding Protein 1 (IGFBP1) [4]. Fasting for 3 days or more has been shown to result in a 30% or greater reduction in circulating insulin and glucose levels and IGF-1 [43]. In our study, with 6 weeks of 8 h TRF treatment, a significant decrease was detected in fasting insulin, fasting blood glucose, HbA1c, LDL-C, triglyceride levels and HOMA-IR score, and a significant increase in TSH and HDL-C levels. In one study, a 6-week program of 8 h TRF diet in women with anovulatory PCOS was investigated, and was found to yield significant improvements in fasting insulin, HOMA-IR, and IGF-1; however, no improvements were observed for glucose level and lipid profile [13]. In the animal study of Han et al., it was shown that mice with PCOS treated with 8 weeks of TRF had healthier glucolipid metabolism compared to controls [15]. Of note, some previous studies reported uncertain results regarding the effects of TRF diets on plasma fasting glucose and lipid levels [21,22]. Harvie et al. reported that the 6-month 5:2 diet and the continuous energy restriction diet improved lipid profile (albeit non-significantly) and insulin resistance in premenopausal overweight women [36]. Results of several studies suggest that Ramadan fasting reduces insulin resistance and glucose levels and improves lipid profile, reducing risks for chronic diseases [44,45]. However, it is not clear whether Ramadan-like fasting could have a significant effect on these parameters in PCOS—especially if patients fail to lose weight [14]. In the few available studies, there is insufficient evidence for the positive effects of alternate day fasting on glucose homeostasis, metabolic regulation and cardiovascular health [16,46]. It is known that TSH is a hormone that shows a circadian rhythm [47]. Independent of PCOS, TSH has been shown to increase after ketogenic diet [47]. In our study, it was shown that TSH increased with TRF diet in patients with PCOS. The reason for this result may be the fasting hours, but this argument needs to be supported by comprehensive studies. Clinical studies evaluating the impact of IF in PCOS are extremely limited. We therefore believe that our study is an important addition to the literature, particularly with respect to its direct assessment regarding PCOS; however, before TRF can be recommended as a therapeutic option, the risks and pitfalls of the diet and its application must be ascertained and accounted for with comprehensive studies.

Some researchers have suggested that the appearance of insulin resistance may result from dysbiosis of the gut microbiota [24,26,48]. Lindheim et al. reported low diversity in the stool microbiome of PCOS women [48]. Tremellen et al. put forward a hypothesis known as “dysbiosis of the gut microbiota” that may partially explain PCOS pathogenesis [26]. As per this hypothesis, adhering to a low-fiber, high-sugar and high-fat diet can disrupt gut microbiota, increasing intestinal permeability. Consequently, circulatory lipopolysaccharides increase in blood, triggering the immune system, ultimately resulting in insulin resistance [26]. Calprotectin binds calcium and zinc and exhibits antimicrobial properties [49]. Fecal calprotectin, therefore, can be used to gauge inflammation and permeability in the gut [48,50]. In one study, it was observed that serum calprotectin was significantly elevated in patients with PCOS relative to controls, and calprotectin levels were correlated with insulin resistance [49]. We observed that the TRF diet reduced fecal calprotectin levels. This is an important finding linking intestinal permeability, PCOS and insulin resistance, which are relationships that have been suggested previously—but without direct evidence [49]. Our study shows considerable changes in fecal calprotectin levels and provides evidence that calprotectin may be an important contributor to the relationships between PCOS, TRF and insulin resistance.

Our research presents significant insights into the effectiveness of the TRF diet as an initial treatment for PCOS patients. However, there are certain limitations associated with our study. Firstly, it is a retrospective study with a relatively small sample size that did not include a control group (without diet or on a different diet). Furthermore, the diet duration was limited to six weeks, and as a result, we were unable to determine the outcomes of a more extended period of dieting or the optimal duration of the TRF diet. The exclusion criteria were set extensively to minimize the effects of external factors on the investigated parameters (for example, patients aged <18 and >40 years or with a BMI of >30 or <18 kg/m^2^), but this also limited the generalizability of our findings. Moreover, various parameters related to inflammation that may impact glucose/lipid metabolism, obesity, and hyperandrogenism were not examined. The effects of diet on menstrual cycle anomalies and ovulatory results, medium- and long-term complications of TRF, and differences in effects for different PCOS phenotypes [4] were not investigated.

## 5. Conclusions

In conclusion, a 6-week program of 8 h TRF provided significant improvements in anthropometric, hormonal, and metabolic (especially insulin–glucose and lipid) profiles in women with PCOS. TRF therapy appears to be a suitable IF protocol that can be used as a treatment of choice in PCOS. However, comprehensive trials and longitudinal studies are needed to demonstrate its efficacy, safety, and superiority over other dietary protocols.

## Figures and Tables

**Table 1:** Summary of patients’ characteristics and laboratory measurements before and after 8 h time-restricted feeding diet.

	Before	After	*p*
Age	25.57 ± 2.67	-	-
Body mass index (kg/m^2^)	25.12 ± 3.17	22.13 ± 2.06	<0.001
Waist to hip ratio	0.85 (0.82–0.88)	0.82 (0.82–0.83)	0.001
Calprotectin (µg/g)	81.5 (13–267)	31 (9–78)	<0.001
AMH (ng/mL)	4.17 (2.76–5.72)	2.66 (2.34–3.3)	<0.001
FSH (mlU/mL)	5.11 (4.1–6.45)	4.52 (3.6–5.18)	0.002
LH (mlU/mL)	9.95 (6.7–11.7)	5.23 (4.32–6.33)	<0.001
E2 (mlU/mL)	43 (35–52)	33 (30–39)	<0.001
TSH mlU/mL	1.11 (0.8–1.76)	1.68 (1.23–1.93)	0.001
Prolactin (ng/mL)	21 (17.3–24.9)	19 (17–21)	0.038
Fasting insulin (μU/mL)	20.17 (11.3–26.4)	13.5 (10.3–17.8)	<0.001
Fasting blood glucose (mg/dL)	89.33 ± 8.39	83.17 ± 5.93	<0.001
HOMA-IR	4.29 ± 1.82	2.87 ± 0.98	<0.001
Insulin resistance (>2.4)	25 (83.3%)	19 (63.3%)	0.070
HbA1c	5.27 ± 0.42	4.96 ± 0.34	<0.001
Total testosterone (ng/dL)	53.34 (25.48–77.15)	32.98 (13.18–41.57)	<0.001
Free testosterone (pg/mL)	1.75 (0.86–2.12)	0.78 (0.58–1.05)	<0.001
SHBG (nmol/L)	43.6 (33.5–66.9)	76.4 (57.4–86.7)	<0.001
Free androgen index	3.92 (1.32–7.56)	1.46 (0.53–2.58)	<0.001
Hyperandrogenism (≥8)	7 (23.3%)	0 (0.0%)	0.016
DHEAS (µg/dL)	252.95 (186.3–296.1)	188.05 (167.8–213.4)	<0.001
HDL-C (mg/dL)	47.2 (41.9–62.2)	67.25 (56.3–73.7)	<0.001
LDL-C (mg/dL)	136 (84–158)	97 (77–117)	<0.001
Triglyceride (mg/dL)	170.5 (135–212)	120 (94–143)	<0.001

Data are given as mean ± standard deviation or median (1st quartile–3rd quartile) for continuous variables according to normality of distribution and as frequency (percentage) for categorical variables. Abbreviations: AMH: Anti-Mullerian hormone, BMI: Body mass index, DHEAS: Dehydroepiandrosterone sulfate, E2: Estradiol, FSH: Follicle-stimulating hormone, HbA1c: Glycated hemoglobin, HDL-C: High-density lipoprotein cholesterol, HOMA-IR: Homeostatic model assessment-insulin resistance, LDL-C: Low-density lipoprotein cholesterol, LH: Luteinizing hormone, SHBG: Sex hormone-binding globulin, TSH: Thyroid-stimulating hormone, WHR: Waist-to-hip ratio.

## Data Availability

The data that support the findings of this study are available from the corresponding author, upon reasonable request.
